# MRI-Monitored Intra-Shunt Local Agent Delivery of Motexafin Gadolinium: Towards Improving Long-Term Patency of TIPS

**DOI:** 10.1371/journal.pone.0057419

**Published:** 2013-02-28

**Authors:** Han Wang, Feng Zhang, Yanfeng Meng, Tong Zhang, Patrick Willis, Thomas Le, Stephanie Soriano, Erik Ray, Karim Valji, Guixiang Zhang, Xiaoming Yang

**Affiliations:** 1 Department of Radiology, Shanghai First People’s Hospital, Shanghai Jiao Tong University School of Medicine, Shanghai, China; 2 Image-Guided Bio-Molecular Interventions Section, Department of Radiology, Institute for Stem Cell and Regenerative Medicine, University of Washington School of Medicine, Seattle, Washington, United States of America; 3 Department of Radiology, Sir Run Run Shaw Hospital, Zhejiang University School of Medicine, Hangzhou, China; Kaohsiung Medical University Hospital, Kaohsiung Medical University, Taiwan

## Abstract

**Background:**

Transjugular intrahepatic portosystemic shunt (TIPS) has become an important and effective interventional procedure in treatment of the complications related to portal hypertension. Although the primary patency of TIPS has been greatly improved due to the clinical application of cover stent-grafts, the long-term patency is still suboptimal. This study was to investigate the feasibility of using magnetic resonance imaging (MRI)-monitored intra-shunt local agent delivery of motexafin gadolinium (MGd) into shunt-vein walls of TIPS. This new technique aimed to ultimately inhibit shuntstenosis of TIPS.

**Methodology:**

Human umbilical vein smooth muscle cells (SMCs) were incubated with various concentrations of MGd, and then examed by confocal microscopy and T1-map MRI. In addition, the proliferation of MGd-treated cells was evaluated. For in vivo validation, seventeen pigs underwent TIPS. Before placement of the stent, an MGd/trypan-blue mixture was locally delivered, via a microporous balloon, into eleven shunt-hepatic vein walls under dynamic MRI monitoring, while trypan-blue only was locally delivered into six shunt-hepatic vein walls as serve as controls. T1-weighted MRI of the shunt-vein walls was achieved before- and at different time points after agent injections. Contrast-to-noise ratio (CNR) of the shunt-vein wall at each time-point was measured. Shunts were harvested for subsequent histology confirmation.

**Principal Findings:**

In vitro studies confirmed the capability of SMCs in uptaking MGds in a concentration-dependent fashion, and demonstrated the suppression of cell proliferation by MGds as well. Dynamic MRI displayed MGd/blue penetration into the shunt-vein walls, showing significantly higher CNR of shunt-vein walls on post-delivery images than on pre-delivery images (49.5±9.4 vs 11.2±1.6, *P*<0.01), which was confirmed by histology.

**Conclusion:**

Results of this study indicate that MRI-monitored intra-shunt local MGd delivery is feasible and MGd functions as a potential therapeutic agent to inhibit the proliferation of SMCs, which may open alternative avenues to improve the long-term patency of TIPS.

## Introduction

Since it was first introduced into clinical practice in 1980s, the transjugular intrahepatic portosystemic shunt (TIPS) has become an important and effective interventional procedure in the management of complications related to portal hypertension [1]–[4]. Although the primary patency of TIPS has been greatly improved as its techniques improved (especially by the application of covered stent-grafts), the long-term patency of TIPS is still suboptimal [5]–[8].

The causes of TIPS dysfunction are multifactorial [9]. The major reasons for limited long-term patency of TIPS are acute occlusion due to thrombosis and the development of pseudointimal hyperplasia in the TIPS or intimal hyperplasia in the hepatic venous outflow [7], [9]–[12]. Histologic studies have confirmed that either pseudointimal or true intimal hyperplasia results from migration and proliferation of venous smooth muscle cells (SMCs) sometimes related to cellular injury when the track is exposed to bile [9], [11], [12].

Several studies have also demonstrated that the hepatic venous outflow is the primary site of stenosis when covered stent are used to construct the TIPS [13], [14]. To overcome this problem, the current practice is to extend the outflow ends of the stent-grafts from the TIPS tract into inferior vena cava (IVC). However, since TIPS is sometimes performed as a bridge to orthotopic liver transplantation (OLT), surgeons prefer that the position of the stent-graft should not interfere with subsequent transplantation [15]–[18]. The current clinical practice of OLT basically involves two types of approaches: conventional OLT and piggyback OLT [16], [19]. For the conventional OLT approach, since the recipient’s retrohepatic IVC is resected together with the native liver during the hepatectomy [16], [19], extending a stent-graft from the TIPS tract into IVC of the bridged recipient should not interfere with subsequent OLT surgery. In contrast, for the piggyback OLT approach, the donor liver is transplanted with a short segment of the donor IVC, which is then directly anastomosed to the main hepatic vein confluence of the recipient [20]–[22]. Since the piggyback technique is the most widely used liver transplant surgery around the world to date [19], it is ideal for OLT that the main hepatic veins of the bridged recipients are preserved with no extension of the TIPS stent-grafts into IVC.

Motexafin gadolinium (MGd), a gadolinium texaphyrin analog, is primarily designed as an intracellularly localized radiosensitizer [23]. Its mechanism of action is oxidation of intracellular reducing molecules and acting as a direct inhibitor of mammalian ribonucleotide reductase (RNR), which is the the enzyme responsible for maintaining a balanced supply of deoxyribonucleotides (dNTPs) required for DNA synthesis and repair and playing a critical role in cell proliferation [24]. Moreover, MGd can produce red fluorescence when stimulated by blue light, and, thus, can be tracked under fluorescence microscopy [25]. Because MGd contains gadolinium, it can also function as a T1 magnetic resonance imaging (MRI) contrast agent [26], [27]. These characteristics of MGd encouraged us to use it as a promising agent for both MRI-monitored intra-shunt local MGd delivery and inhibiting the proliferation of venous SMCs.

To address above mentioned clinical problem, we explored a novel method to prevent hepatic venous outflow stenosis following TIPS creation. In this study, we investigated a technique of MRI-guided intra-shunt local agent delivery of MGd, which aimed to ultimately inhibit post-TIPS stenosis. The main purpose of this study was to validate the feasibility of using MRI to monitor intra-shunt local delivery of MGd into shunt-vein walls of TIPS.

## Materials and Methods

### Study Design

**Figure 1:**
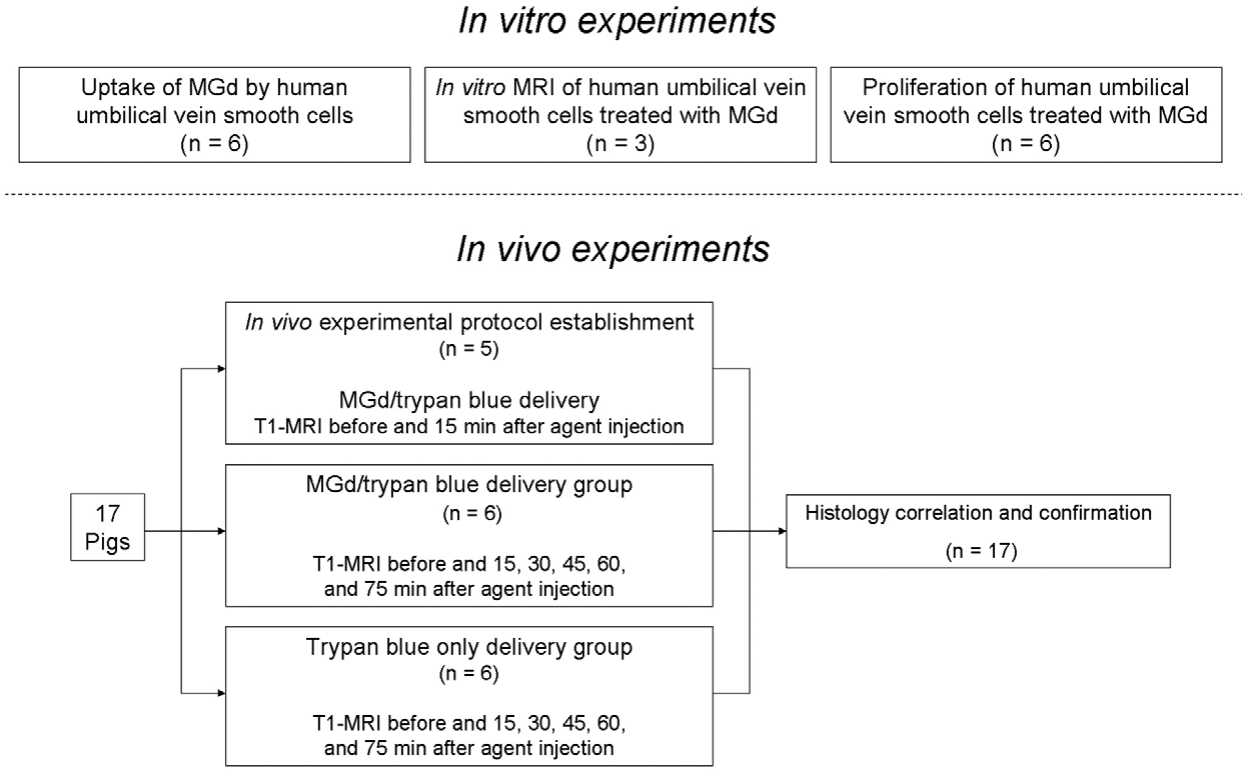
Study design.

This study was divided into two phases (Fig. 1): *(a)* in vitro experiments to confirm the intracellular uptake of MGd by human umbilical vein smooth muscle cells; and *(b)* in vivo experiments to validate the feasibility of MRI-monitored intra-shunt local MGd delivery.

### In vitro Experiments

#### Cell culture

Human umbilical vein smooth muscle cells, which were primary human venous SMCs derived from tunica media of a healthy umbilical vein, were purchased from Cell Applications, Inc. (San Diego, CA). These cryopreserved cells were thawed and plated according to the manufacturer instruction, and then were cultured with the smooth muscle cell growth medium (Cell Applications, Inc., San Diego, CA) at 37°C and 5% CO_2_ in a humidified incubator (Hera Cell 150, Thermo Scientific, Waltham, MA).

#### Cellular uptake of MGd

The first group of SMCs was incubated with MGd (Pharmacyclics, Inc., Sunnyvale, CA) at various concentrations of 0, 25, 50, 75, 100, 125, and 150 µg/mL for 6 hours. The treated cells were then washed three times with phosphate-buffered saline (PBS) to remove free MGd, and thereafter fixed in 4% paraformaldehyde. The cells were counterstained with 4′,6-diamidino-2-phenylindole (DAPI, Vector Labroatories, Inc., Burlingame, CA), and then imaged with a laser confocal microscope (A1R, Nikon Inc., Tokyo, Japan) to compare the intensities of MGd-emitted red fluorescence in the plasma of different groups of cells.

#### In vitro MRI experiments

To determine the optimal dose range of MGd, the second group of SMCs were cultured and grown on 6-well plates (BD Falcon, Franklin Lakes, NJ). The subsequent confluent cell cultures were treated with MGd concentrations of 0, 25, 50, 75, 100, 125, and 150µg/mL for 6 hours. The cells were washed three times with PBS to remove free MGd, and then were counted using an automated cell counter (TC10, Bio-Rad Laboratories, Inc., Hercules, CA). Approximately 1×10^6^ cells were then transferred to 0.1-mL glass tubes and dispersed in 0.5% agarose. After this, the tubes were placed into a custom-designed holder, in which 1% agarose was filled to exclude the air. MRI of these cell-containing tubes was obtained on a 14.7 Tesla MR system with a 25-mm diameter coil (Utrashield 600WB Plus, Bruker, Billerica, MA). T1-map images were acquired in the transverse planes using 800/9.7-msec repetition time (TR)/echo time (TE), 180° flip angle, 25.6-mm field of view (FOV), 1-mm section thickness with 0-mm intersection gap, 256×128 matrix, 7 number of T1 experiments, 1 number of repetitions, and 1 number of echo images.

#### Imaging analysis of in vitro MRI experiments

T1-map MRI of SMCs with various concentrations of MGd was also analyzed by measuring the T1 values of each cell-containing tube. T1 values of these tubes were then recorded by using the software provided by the MR manufacturer. A region of interest (ROI) at 1 mm^2^ size was placed at the center of each tube. The 1 mm^2^ ROI was placed by one radiologist (H.W., 12 years of experience with MRI).

#### MTS assay for evaluating the proliferation of SMCs treated by MGd

The third group of SMCs was specifically designed for MTS [3-(4,5-dimethylthiazol-2-yl)-5-(3-carboxymethoxyphenyl)-2-(4-sulfophenyl)-2H-tetrazolium] assay. The cells were treated with MGd at concentrations of 0, 25, 50, 75, 100, 125, and 150 µg/mL in 96-well plates (1×10^4^ cells/well) for 6, 12, 18, 24, 36, and 48 hours, respectively. The treated cells were washed three times with PBS to remove free MGd. Then, 20-µL CellTiter 96 Aqueous One Solution Reagent (Promega, Madison, WI) with its 100-µL medium were added to each well. The plates were continuously incubated at 37°C in a humidified, 5% CO_2_ incubator for 2 hours. According to the manufacturer instruction, the absorbance of MGd-treated cells was then measured at 490 nm using a plate reader (Spectra max M2, Molecular Devices, Sunnyvale, CA).

### In vivo Experiments

#### Animals

Animal experiments were designed in compliance with National Institute Health Guideline for the Care and Use of Laboratory Animals, and the animal protocol was approved by the Institutional Animal Care and Use Committee of University of Washington (Approval ID 4120-02). Seventeen domestic pigs (weight range 40–60 Kg) were obtained from Washington State University (Washington, WA) and all 17 pigs underwent TIPS procedures. The pigs were divided into three groups: (1) five pigs were used to establish the experimental protocol; (2) six pigs were subjected to local delivery of MGd mixed with trypan blue solution (Amresco Inc., Solon, OH) into the shunt-hepatic vein walls; and (3) six pigs were locally delivered with trypan blue-only to serve as controls.

#### TIPS procedure

**Figure 2:**
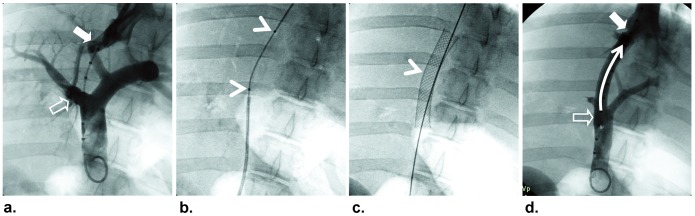
Main steps of the TIPS procedure with local MGd delivery into the shunt-vein wall. Simultaneous venograms show the location and length of the shunt between the right portal vein (open arrow) and the right hepatic vein (closed arrow) (a). Placement of the custom-made microporous balloon for the agent delivery into the shunt-vein wall. Arrowheads indicate the two markers of the balloon (b). A deployed stent (arrowhead) through the TIPS shunt (c). Post-procedure venogram shows that the contrast in the main portal vein (open arrow) is flowing though TIPS shunt (curved arrow) into the hepatic vein (closed arrow) (d).

All pigs were first sedated by intramuscular injection of Telazol at 4.4 mg/kg body weight and Xylazine at 1 mg/kg body weight, and then mechanically ventilated with 1–3% isoflurane. The pigs subsequently underwent TIPS procedures in an angiography suite. Briefly, the right jugular vein was accessed by a 4-F micro-puncture set (MPIK-4-SP7, B. Braun Medical Inc., Bethlehem, PA) under ultrasound guidance. A 10-F TIPS set (RUPS-100, Cook Medical., Bloominton, IN) was then advanced, via the superior vena cava and the right atrium of the heart, into a hepatic vein. Once successful puncture of the portal vein was achieved, a 260-cm-long, 0.035-inch steerable guide wire (Back-up Meier, Boston Scientific, Miami, FL) was advanced into the superior mesenteric vein (SMV). After placing a 90-cm-long, 5-F gold marker pigtail catheter (Royal Flush, Cook Medical, Bloominton, IN) in the SMV and a 40-cm-long, 10-F TIPS sheath into the hepatic vein over the guidewire, simultaneous venograms were obtained by (i) power injection of 14-mL nonionic contrast medium (iohexol [Omnipaque], 300 mg of iodine per milliliter, GE Healthcare Inc., Princeton, NJ) at 7 mL/sec for three seconds using an automatic injection system (Stellant Dual, Medrad Inc., Pittsburgh, PA) from the pigtail catheter; and (ii) manual injection of 15-mL contrast agent at 50% concentration at approximately 5 mL/sec for three seconds. The simultaneous venograms enabled us to determine the length of the created parenchymal tract (shunt) between the hepatic vein and the portal vein. Then, a 6 mm×4 cm custom-made microporous delivery balloon catheter was placed into the shunt with the center of the balloon just across the junction of the hepatic vein to the shunt. The MGd delivery balloon catheter consisted of an angioplasty balloon with multiple micropores on the balloon surface. The inflation of the balloon with MGd stopped the blood flow completely in the vessel and simultaneously propelled the micropores against the targeted vessel wall. Thus, subsequent infusion of the MGd caused, via an orifice-mediated jet effect, the disruption of the physical barriers imposed by the continuous endothelium and the internal elastic lamina, and, thereby, the delivery of MGd into the media of the targeted vessel wall. Figure 2 shows the main steps of the TIPS procedure, with local delivery of MGd into the shunt-vein walls.

#### MRI monitoring for the intra-shunt local agent delivery

The pig was then transferred to a 3 Tesla MR scanner (Achieva 3.0 T TX, Philips Healthcare, Best, the Netherlands). Each pig was placed supine on the MR scanner table. We first inflated the microporous balloon with 1.5-mL saline, which enabled us to easily localize the inflated balloon as hyperintensive signal on MRI. To this end, we acquired fat-suppressed and respiration-gated T2-weighted images with SE sequence using a Torso phase-array surface coil to locate the shunt. Before MGd/blue delivery, axial and coronal T1-weighted images were obtained with SE sequence. Then, 20-mL MGd (100 µg/mL) diluted with trypan blue solution or 20-mL trypan blue solution only (for control pigs) was infused through the microporous balloon into the shunt-vein wall at 1 mL/sec flow rate under dynamic contrast-enhanced MRI (DCE-MRI) monitoring using a gradient echo sequence with 36 seconds breath holding. After the completion of the agent delivery, the axial and coronal T1-weighted images using the same parameters as used pre-agent delivery was achieved again. For the post-agent delivery MRI, the first group of five pigs for establishment of the experimental protocol was scanned only one time at 15 min after agents injection, while the second and third groups of 12 pigs were scanned over time at 15, 30, 45, 60, and 75 minutes after each agent injection. The parameters of T1 weighted images pre- and post-agent deliveries included: 1400–3500/5-msec TR/TE, 250-mm FOV, 156×156 matrix, 90° flip angle, 3-mm section thickness with 1-mm intersection gap, and five signals acquired. The DCE-MRI was scanned with the following parameters: 126/5-msec TR/TE, 220-mm FOV, 144×141 matrix, 50° flip angle, 5-mm section thickness with 2-mm intersection gap, seven signals acquired, and 2 phases.

#### Imaging analysis

We measured signal intensities (SI) of the shunt walls (SI_shunt_) and the off-shunt liver parenchyma (SI_off-shunt_) using four ROIs along the shunts at 0, 3, 6, and 9 o’clock, and then calculated the average contrast-to-noise ratio (CNR) using (SI_shunt_-SI_off-shunt_)/standard deviation of the background noise (SD_noise_). The 1 mm^2^ ROI was placed by one radiologist (H.W., 12 years of experience with MRI).

#### Stent deployment

After MRI, the pig was transferred back to the angiography suite. Because the main aim of the present study was validating the MRI-monitored local MGd delivery technique, we did not try to use the expensive covered (Viatorr) stent-graft for all shunt creation. An 8-mm-diameter, 6-cm-long Wallstent (Unistep Plus, Boston Scientific, Miami, FL) was deployed through the shunt from the right portal vein to the hepatic vein. Then, a 10 mm×4 cm balloon catheter (PowerFlex, Cordis, Miami, FL) was used to dilate the Wallstent. Finally, the pigtail catheter was placed though the stented shunt into the SMV to obtain post-TIPS venography.

#### Histology

Immediately after post-TIPS venography, each animal was euthanized by intravenous injections of pentobarbital sodium (100 mg/kg). Each of the stented shunts along with the hepatic and portal vein segments was harvested for histologic correlation to confirm the successful penetration of MGd/trypan blue into the shunt-vein wall. We cryosectioned the specimens at 5-µm thinkness, and then examined the histologic slides by using *(a)* laser confocal microscopy (A1R, Nikon Inc., Tokyo, Japan) to detect red-fluorescent MGd; and *(b)* light microscopy (BX51, Olympus, Tokyo, Japan) to detect Trypan-blue staining of the shunt-vein walls.

### Statistical Analysis

SPSS version 17.0 (SPSS Inc, Chicago Ill) was used to perform the statistical analyses, and a Student t-test was used to compare the average CNRs between the pre- and post-agents delivery in each animal study group. All data are presented as mean±standard deviation. A *P* value less than 0.05 was considered statistically significant.

## Results

**Figure 3:**
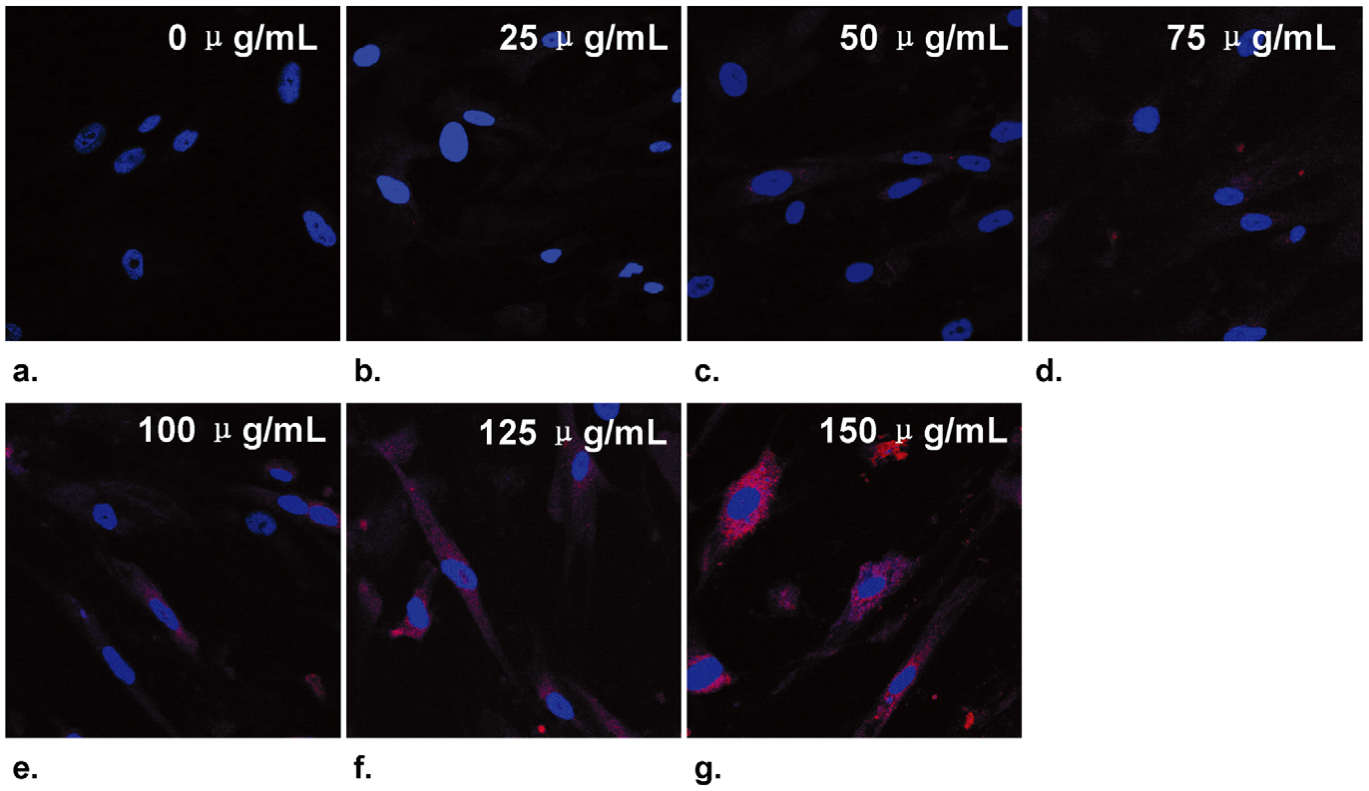
Confocal microscopic images of SMCs incubated with MGd at various concentrations of 0 to 150 µg/mL. The images demonstrate that an increased intracellular uptake of MGd in cytoplasm (red fluorescent dots) as MGd concentration increases.

**Figure 4:**
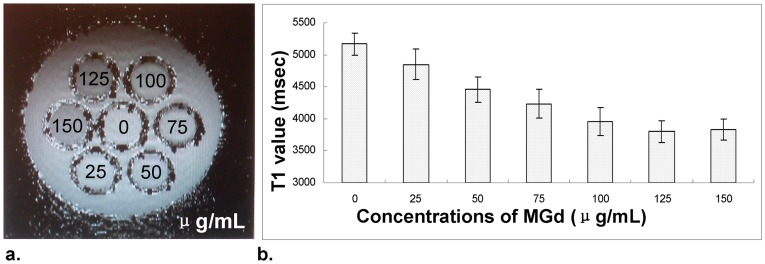
In vitro 14.7T MR T1-map images and the relative T1 values of SMCs incubated with MGd at various concentrations. The T1-map images show the decreased bright signals as MGd concentration increases (a). Measurement of T1 value further confirms the findings in **a**, demonstrating a linear decrease of T1 value from 0–100 ug/mL MGd, followed by a platform pattern of T1 values from 100–150 ug/mL MGd (b).

**Figure 5:**
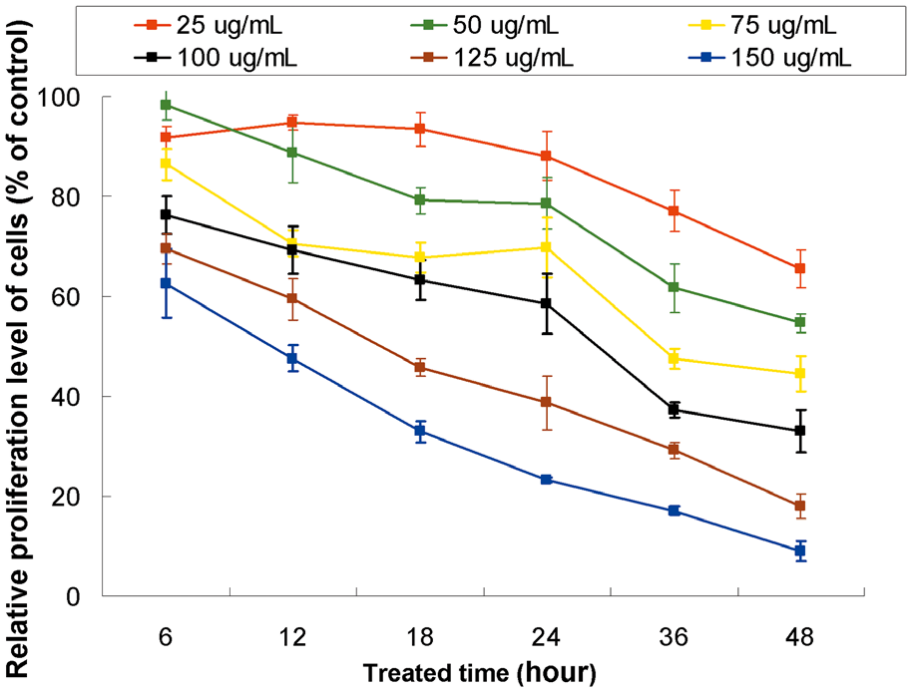
Proliferation of SMCs treated with MGd at various concentrations. The proliferation levels decrease as MGd concentrations increase and as the treated times increase.

In the *in vitro* experiments, confocal microscopy confirmed the intracellular uptake of MGd by SMCs, showing an increased MGd uptake as the MGd concentrations increased (Figure 3). *In vitro* T1-map MRI further confirmed these cytologic findings, demonstrating a linear decrease of T1 value from 0 to 100-mg/mL and then a platform pattern of T1 values from 100 to 150 mg/mL of MGd (Figure 4). MTS assay showed that the proliferation levels of SMCs decreased as the MGd concentrations increased and as the MGd-treating times increased (Figure 5).

**Figure 6:**
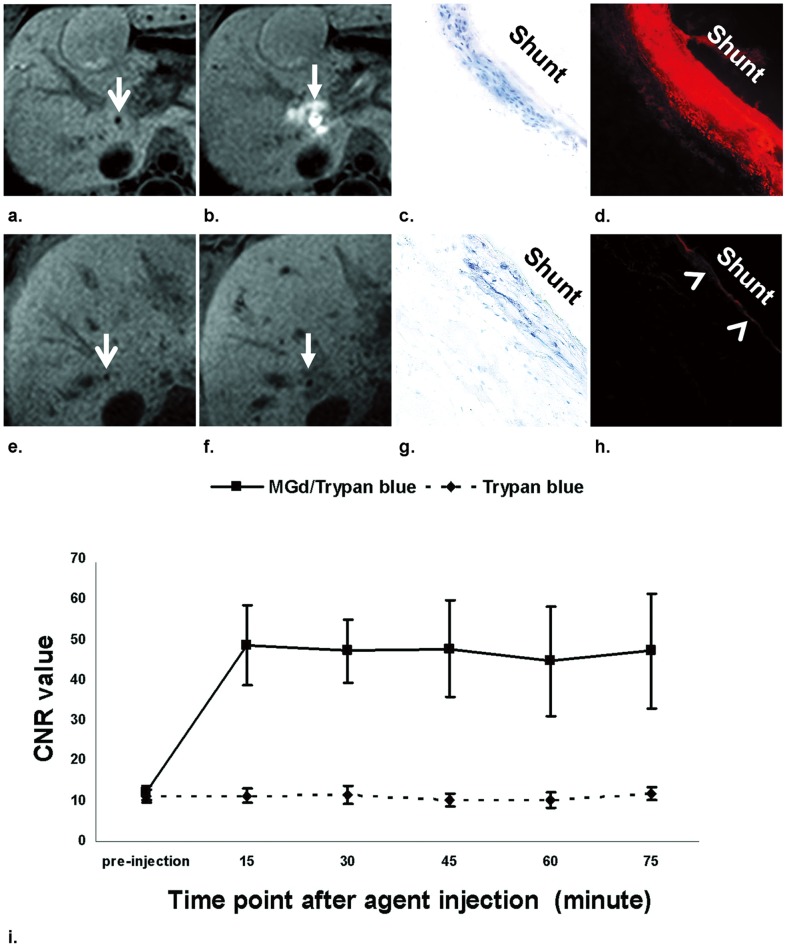
In vivo MRI with subsequent histology confirmations of intra-TIPS delivery of MGd/trypan-blue mixture (a–d) and trypan-blue only (e–h). MRI manifests the TIPS shunts before intra-TIPS local deliveries of MGd/blue mixture (arrow on a) and trypan-blue only (arrow on e), and the MGd distribution around the shunt (arrow on b) as bright signal after intra-TIPS agent delivery of MGd/blue, which is not seen on MRI with intra-TIPS delivery of trypan-blue only (arrow on f). Histological images confirm the successful penetration of trypan-blue (as blue stain on c) and MGd (as red fluorescent spots on d) in the shunt - hepatic vein wall with delivery of MGd/blue, while in the control group with delivery of trypan-blue (g and h), only trypan-blue stain (g) is visualized within the shunt - hepatic vein wall with weak autofluorescence of the intima (arrowhead on h). (Original magnification, ×20). Time-CNR curves of each animal group (i), showing that CNR increases immediately after MGd/blue delivery and remains at a high level within 75 minutes in the MGd/blue-treated group, while in the control group, the CNR does not increase after the trypan blue-only delivery.

Figure 6 summarizes the results of the *in vivo* dynamic MRI monitoring of local agent deliveries during the TIPS procedure with subsequent histology confirmations. In the animal study group with intra-shunt delivery of MGd/blue mixture, MRI displayed the bright enhancement around the TIPS tract due to MGd/blue penetration into the shunt-vein walls, which was not seen in the control pigs. Histological findings further confirmed the successful penetration of blue-dye (as blue stain) and MGd (as red-fluorescent spots) within the shunt - hepatic vein walls in the animal group with MGd/blue, while in the control group, only blue stain was visualized in the shunt-vein wall with weak auto-fluorescence of the intima. Moreover, compared to the control group, the MGd/blue-delivered animal group demonstrated significantly higher CNR of shunt-vein walls on post-delivery MR images than on pre-delivery images (49.5±9.4 vs 11.2±1.6, *P*<0.01). The longitudinal observation demonstrated that the CNR increased immediately and remained continuously at a high level within 75 minutes after the MGd/blue delivery, while in the control group, the CNR did not increase after the blue-only delivery.

## Discussion

With covered devices, the hepatic venous outflow of the TIPS tract is the major site of subsequent obstruction [14], [28]. Although the clinical application of covered stent-grafts has improved the primary patency rates after TIPS procedures [5], [6], the one-year patency was reported to be still limited, even as low as 38% [29]. In the present study, we specifically focused on the site of the hepatic venous outflow to develop a technique using MRI to monitor the local delivery of therapeutic agents to the shunt-vein walls and thereby improve the long-term patency of TIPS.

MRI-guided intervention offers several important advantages [30], [31]. First, MRI affords superior soft tissue distinction compared with other imaging modalities. Second, it allows multiplanar three-dimensional reconstructions and maximum intensity projections with sufficiently good spatial resolution to guide interventions. Third, real-time MRI can be used to guide the procedure and monitor the drug delivery during the intervention itself. Fourth, it avoids radiation exposure compared to conventional X-ray angiography-guided interventions. Thus, in the present study, we selected MR to be the imaging modality to monitor intra-shunt local delivery and distribution of agents during the TIPS procedure.

For monitoring of the agent delivery and distribution, application of MRI contrast agents is essential. MGd is a porphyrin-like molecule that is taken up preferentially by metabolic active cells, such as SMCs. MGd is useful as *(a)* a chemotherapeutic agent [23], *(b)* a T1-weighted MRI contrast agent [26], [27], and *(c)* an emitter of red-colored fluorescence for histologic/laboratory correlation [25]. Because the results of a pilot study demonstrated the 3 T MRI could not offer satisfactory detection sensitivity for the cellular T1-mapping of MGd-treated SMCs, we specifically used a 14.7 T MR system to investigate the T1 relativity time of the cells treated with MGd at various concentrations. The results confirmed the effect of MGd to shorten the T1 value of the treated cells in a concentration-dependent fashion. In the present in vivo study, we used a clinic 3 T MR system and a very low dose of MGd, which enabled us to validate the feasibility of using MRI to not only monitor the distribution of MGd in the shunt-vein walls, but also quantify the CNR changes of the shunt-vein walls before and after MGd delivery. Moreover, this study demonstrates infiltration of MGd into the shunt-vein wall tissue that kept the CNR at a high level as long as 75 minutes after the MGd/blue treatments. This observation indicates that MGd was taken into the cells of the shunt-vein wall tissue, which could allow monitoring of uptake of an agent with potential to limit post-TIPS stenosis.

The proliferation of SMCs is believed to be the dominant cause of the pseudointimal hyperplasia responsible for stenosis of the TIPS tract [9], [11], [12]. It had been clarified that MGd inhibits the proliferation of cell by inhibiting the cellular RNR [24]. The results of our study demonstrated that MGd could be efficiently taken up by SMCs and suppressed the proliferation of these cells. These encouraging findings strongly indicate that MGd may be used as a potential therapeutic agent to inhibit the proliferation of SMCs, and therefore can be locally delivered into the shunt-vein walls to inhibit pseudointimal hyperplasia within the TIPS tract.

In conclusion, MRI-monitored intra-TIPS local MGd delivery is feasible. MGd may function as a potential therapeutic agent to inhibit the proliferation of SMCs. This technical development may open alternative avenues to improve the long-term patency of TIPS by using MRI-integrated drug/gene therapy.
